# Stevens Johnson Syndrome Initiated by an Adverse Reaction to Trimethoprim-Sulfamethoxazole

**DOI:** 10.7759/cureus.10023

**Published:** 2020-08-25

**Authors:** Adelina Buganu, Massud Atta, Matthew Solomon, Paul R Banerjee, Latha Ganti

**Affiliations:** 1 Emergency Medicine, Coliseum Medical Centers, Macon, USA; 2 Emergency Medicine, Brown University, Providence, USA; 3 Emergency Medicine, University of Central Florida, Orlando, USA; 4 Emergency Medical Services, Polk County Fire Rescue, Bartow, USA; 5 Emergency Medicine, Envision Physician Services, Plantation, USA

**Keywords:** steven johnson syndrome

## Abstract

The authors present a case of a 54-year-old male who presented to the ED with Stevens Johnson syndrome (SJS) beginning on his upper lips, then spreading to his glans penis, airway, and buttocks. After using trimethoprim-sulfamethoxazole (TMP-SMX) to treat a pilonidal cyst diagnosed seven days prior to presentation, the patient began to have desquamating lesions on his upper and lower lips. Subsequently, he noticed desquamation on the glans penis and then between his buttocks. Before being referred to dermatology, he was treated with a high dosage of corticosteroids.

## Introduction

Stevens Johnson syndrome (SJS) is a severe skin disorder that may arise as a reaction from certain medications. A patient suffering from SJS presents a fever, then a red or purple rash that will eventually blister. The blistering portions of the skin usually peel leaving behind a painfully eroded area. SJS can even affect the ears, mucosal surfaces of the mouth, nose, eyes, and airways as well as the genitals and urinary tract. In addition to skin manifestations, patients may develop fevers, myalgias, cough, ptyalism, and dysuria. The skin is a major protective barrier that also helps regulate body temperature with the ability to sweat. SJS is life-threatening because it can severely damage the skin [1].

Stevens Johnson syndrome occurs in 1-2 million people per year [2]. Patients suffering from HIV, Hepatitis A, herpes simplex virus/varicella zoster virus (HSV/VZV) or autoimmune diseases, such as systemic lupus erythematosus, are more likely to experience SJS. Carriers of the gene HLA-B1502 (commonly noted in individuals of southeast Asian, Chinese, and Indian descent) are at an increased risk of developing SJS [3]. Other risk factors include family history of SJS, personal history of SJS, and compromisation of the immune system. SJS is commonly caused by medications such as allopurinol, penicillin, trimethoprim-sulfamethoxazole (TMP-SMX), non-steroidal anti-inflammatory drugs (NSAIDs), and phenytoin among others. In a few reported instances, particularly in pediatric cases, viral infections have been known to cause SJS [1].

Stevens Johnson syndrome is part of the spectrum of skin reactions. Toxic epidermal necrolysis (TEN) is a similar skin blistering disease. SJS and TEN are merely distinguished by the amount of patient body surface area affected by the skin reaction. According to the common classification of SJS and TEN, SJS affects a body surface area of 10% or less. If a patient has skin manifestations of 30% or more, it is likely to be TEN. An area in between 10% and 30% is considered an SJS-TEN combination [1]. Regardless, both diseases are considered dangerous and emergent.

## Case presentation

A 54-year-old male with a previous medical history of hypertension, non-insulin dependent diabetes mellitus, and hyperlipidemia presented to the ED complaining of lip swelling and a rash on his penis. The patient first noticed the swelling on his lip approximately two days prior to presentation. Later, he noticed desquamation of the glans penis. He denied any recent sexual activity and the possibility of a sexually transmitted infection (STI). Further, the patient denied any previous allergic reactions. Approximately seven days prior to presentation, the patient was diagnosed with a pilonidal cyst and was placed on TMP-SMX. He reports adherence with the medication for three days but then he stopped it. The patient restarted the medication the morning his symptoms started.

**Figure 1:**
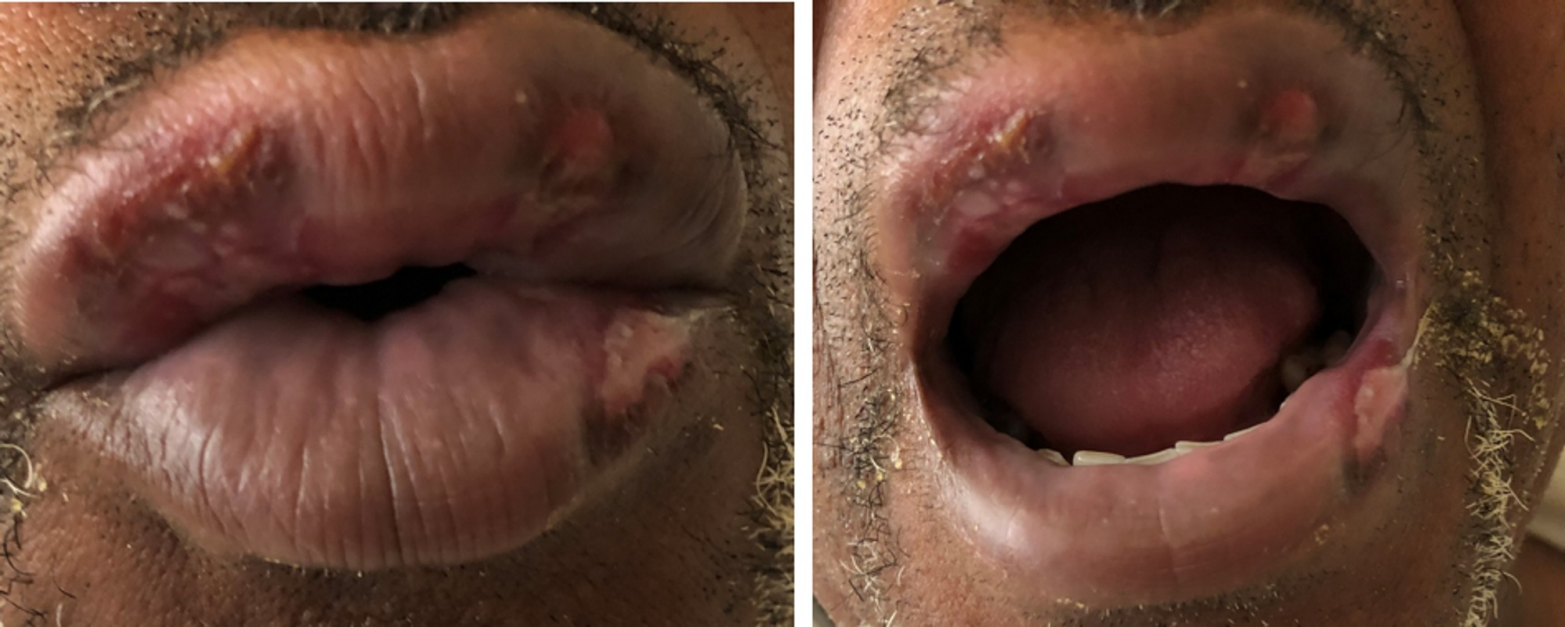
The patient’s upper and lower lips show desquamation caused by SJS. SJS, Stevens Johnson syndrome

His physical examination revealed desquamating lesions on his upper and lower lip associated with swelling in his upper lip (Figure 1).

There were no buccal or ophthalmic lesions present. He did not appear toxic. His vital signs were normal, including heart and respiratory rate. However, he did report a sensation of airway tightness. Laboratory evaluation was suggestive of underlying inflammation with an elevated C-reactive protein count (1.2 mg/dL) (Table 1).

**Table 1:** Patient's laboratory values. BUN, blood urea nitrogen; GFR, glomerular filtration rate; AST, aspartate aminotransferase; ALT, alanine transaminase

Sodium (136-145 mmol/L)	136
Potassium (3.5-5.1 mmol/L)	4.6
Chloride (99-109 mEq/L)	106
Carbon dioxide (22.0-31.0 mEq/L)	26.0
Anion gap (5-15)	8.6
BUN (6-18 mg/dL)	34 H
Creatinine (0.55-1.30 mg/dL)	2.0 L
Est GFR (African American) (>60)	42 L
Est GFR (Non-African American) (>60)	35 L
Glucose (70-100 mg/dL)	229 H
Calcium (8.3-10.4 mg/dL)	9.5
Total bilirubin (0.0-1.0 mg/dL)	0.30
AST (10-37 unit/L)	19
ALT (12-78 unit/L)	37
Alkaline phosphatase (30-136 unit/L)	132
C-Reactive protein (0.05-0.3 mg/dL)	1.2 H
Total protein (6.4-8.2 g/dL)	7.1
Albumin (3.4-5.0 g/dL)	3.5

The patient was admitted for observation due to the potential for airway compromise and treated with a high dose of corticosteroids. He had an uneventful discharge to home. Three days after discharge, he returned to the ED due to persistent symptoms and a new area of desquamation in between his buttocks. He continued with the same treatment of corticosteroids and was subsequently referred to dermatology. 

## Discussion

**Figure 2:**
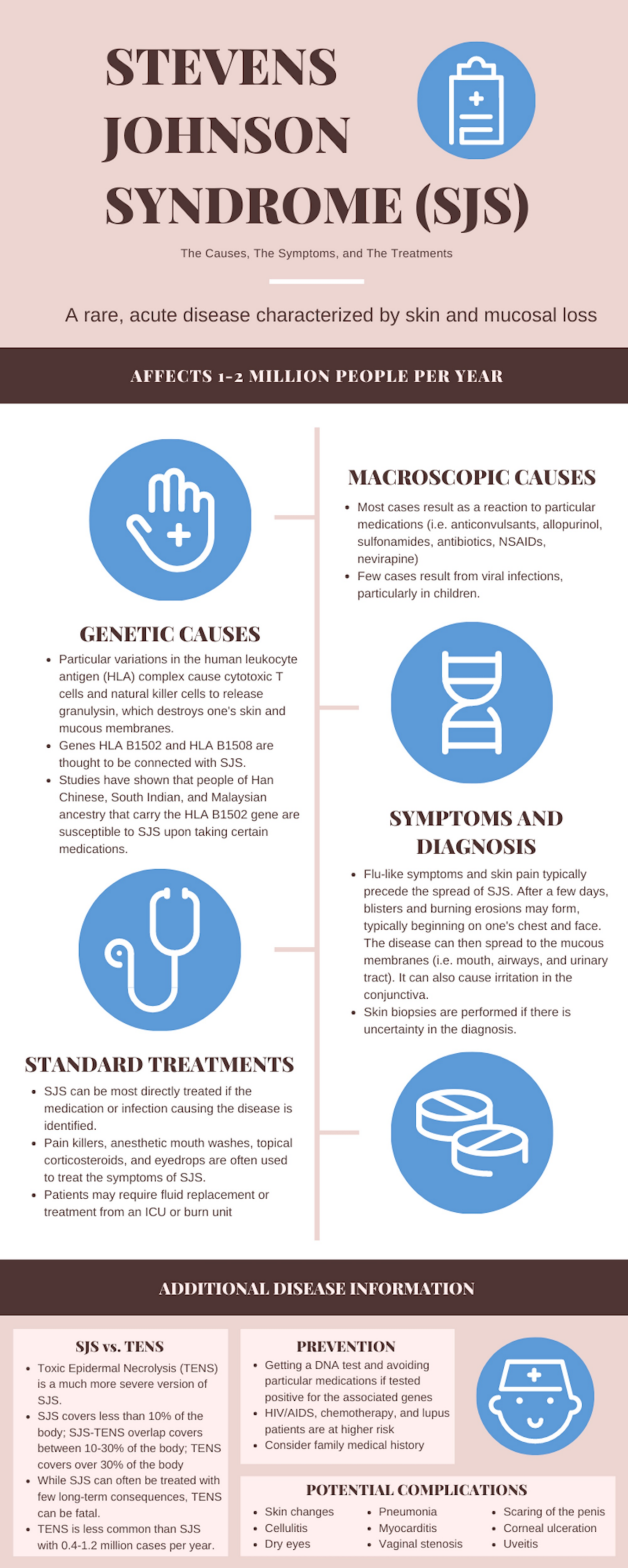
Causes, symptoms, and treatments for SJS (Infographic created by Matthew Y. Solomon). SJS, Stevens Johnson syndrome

This patient’s case of SJS exhibited a widespread distribution of desquamation. Lesions first appeared on the patient’s upper and lower lips, though there was no presentation of buccal or ophthalmic lesions. He then experienced desquamation on the glans penis, subsequently spreading to his airway and then buttocks. As typical of SJS, the affected areas were lined with a mucous membrane. Cases of SJS have been also known to affect the urinary tract and the conjunctiva. Studies have reported that 90% of all cases of SJS affect either the mouth, genital, or gastrointestinal tract [2]. Although not experienced by this patient, it is quite common for SJS patients to experience flu-like symptoms that precede the spread of the disease. Causes symptoms and treatments are summarized in Figure 2.

Stevens Johnson syndrome is widely thought to be associated with particular variations in the human leukocyte antigen (HLA) complex causing keratinocyte apoptosis upon exposure to particular medications [1]. In this process, cytotoxic T lymphocytes and natural killer cells release cytotoxic molecules, mainly granulysin, causing epidermal necrosis [4]. Among the most common medications responsible for this reaction is TMP-SMX, which has accounted for about 20% of cases in several studies [5]. In this case, the patient used bactrim for several days to treat his pilonidal cyst. TMP-SMX is a combination of both trimethoprim and sulfamethoxazole, which makes it likely that this patient’s case of SJS was triggered by this medication. Reports indicate that in order to initiate recovery from the disease, the patient must first stop taking the causative medication.

At the ED, the patient was treated with a high dosage of corticosteroids for airway management and continued symptoms and desquamation before it was confirmed that he had SJS. The use of corticosteroids to treat SJS has been controversial. On one hand, prolonged use of corticosteroids is known to increase the chance of secondary infection. However, a retrospective study at Vajira Hospital in Bangkok, Thailand concluded that a limited short-course of corticosteroids can reduce the mortality rate of SJS without inflicting a secondary infection [6]. Current research is also being done on other potential treatments for SJS. Studies have indicated that cyclosporine is an effective immunomodulator due to it specifically targeting granulysin, halting the dissemination of the disease. Early reports also suggested the efficacy of tacrolimus and cyclophosphamide; however, further studies must be conducted to support these results [7].

## Conclusions

Stevens Johnson syndrome can widely affect the skin and mucosal regions of the body without preceding symptoms. Physicians must be aware that a given medication used to treat one condition may have the potential to cause SJS. As the disease is commonly genetic, physicians must understand their patient’s family medical history with regard to SJS when prescribing medication. Several treatments are currently being studied for more severe cases of SJS-TENS, though the most essential and basic management is to identify and stop using the causative medication.
